# The Value of Illustration in the Lecture Room

**Published:** 1890-08

**Authors:** L. D. M’Intosh


### The Value of Illustration in the Lecture Room.

BY L. D. M'INTOSH, M.D., D.D.S.

The object of this short and hastily written paper is to point out to those who are interested in the work of the lecture room what we believe to be one of the best methods of illustrating the various branches taught.

I think I am safe in making the assertion that teachers in all departments of study see the necessity ot illustrations. Abstract teaching alone, either by lectures or recitation, fails to produce as permanent impression on the student as it does with illustrations. For example, we may give a description of a city, or some place we are familiar with, to a class of students and afterwards ask them to describe the same, we find that no two understand the description as it was given. If views had accompanied the description of the place, showing the streets, buildings, &c., each student would have formed almost as correct an idea as though he had visited the place. We see how attractive and instructive the public lecturer can make his subject, by using views of the scenes he describes. It has become a necessity for him, if he desires to describe his travels, to illustrate them. The public demands this, and enforce their demand by their absence when no illustrations are shown, and, on the other hand, their approval by the large audience present, when his descriptions point to life like scenes. The following illustates the value of illustrated lectures and the permanent impression produced on an audience.

Some 12 years ago I gave a series of popular illustrated lectures in Chicago in my own parish. One lecture was entitled "Microscopic World." In the course of the lecture, a description was given of the structure of a human lung, its capillary net work, congestion, &c., using a microscopic section projected on a screen, so that it could be plainly seen by all of the audience. Many of the people present afterward referred to the illustration saying, although they had studied the subject before, this was the first time it was made clear to them. Only last winter a gentleman, who was present at this lecture, referred to the illustration in this way, he said: "I am just recovering from a severe cold which came near resulting in congestion of the lungs. I imagined I could see the fine net work of blood vessels, in which the blood had ceased to flow and I knew the thing was to get it to circulate again. I remembered what you said about counter-irritation and the application of heat to bring the blood to the surface of the body and thus relieve the congested lung. I produced free perspiration, applied mustard plasters freely and was relieved in a short time." This simply shows the impression produced by the illustrations. We can all remember the lasting impressions produced on us when we were young by the experiments of the itinerant scientific lecturer. I think my love for scientific study was awakened by listening to an illustrated lecture on natural philosophy when I was not more than ten years of age. It was not the words or name of the lecture that I remember, but the experiments which seemed very wonderful to me and are as vivid today as though I had seen them but yesterday.

Some ten years ago I attended a medical society in a neighboring state. An evening was devoted to microscopic illustration, using a projection microscope. The public was invited with the idea to entertain and at the same time show the scientific side of the study of medicine. A large audience was present and all seemed interested. The work of that evening passed from my mind. Some four or five years afterward a physician called on me; in his conversation he asked if I remembered giving microscopic illustrations and assisting Dr. A. at a certain state medical meeting. I told him I did. He said he was present, though not a physician at that time, and those illustrations seemed very wonderful to him. He said until this time he had looked on the profession of medicine indifferently, but what he saw that evening was the first incentive to commence its study. I afterwards learned that this man was an ex-governor.

I am often chagrined and disappointed after carefully writing a description of a scientific intrument and the directions for its use, to find the purchaser has either failed to comprehend them, or they do not give the idea clearly. A well posted physician not long since obtained an instrument for electrical measurement.

After several unsuccessful attempts to use it, he wrote me of his failure. He afterwards brought the instrument to me, and we placed it in proper position with connections, and it worked all right. There was one point in the directions which he did not interpret as it was intended it should be, therefore his failure; but when he saw it connected and used the directions were plain to him.

The use of every scientific instrument and process, to be clearly understood, should be demonstrated and illustrated. Unless illustrations accompany the description, the idea is only partially comprehended. All descriptions in applications for patents must be minutely illustrated in detail, and even then the expert examiners are often obliged to obtain models to aid them. If illustrations are so necessary to aid experts in comprehending descriptions in a certain special line, how much more does the student need the lectures illustrated to which he listens.

In nearly all our schools, from the primary department to the highest grades, the teachers see the necessity of illustrating their work as much as possible, and are constantly devising apparatus to do this more perfectly. In the departments of chemistry, physiology, anatomy, materia medica, microscopy, histology and pathology, operative and prosthetic dentistry, in fact, all scientific study, all teachers use illustrations. Yet, with all that is being done, we believe this work is in its infancy. If there was a greater effort to make a subject plain by illustration, the description cut short, given in few words and to the point, the student would retain what he sees and hears. The scientist and even the office man has caught the spirit of illustration. When they take their vacation, a Kodak camera is their constant companion. By its aid they bring back pictures of the country they have visited to show their friends. Even the clerygman finds he can interest his hearers by giving an illustrated sermon and fill the seats that would otherwise be vacant.

I believe there is no way of illustrating as easily, clearly and cheaply a great part of the lectures in science, medicine and dentistry as by projection. We have at our command very perfect apparatus for this line of work; we can use diagrams, photomicrographs, micro-photographs, microscopic specimens, opaque objects, chemical reactions, crystalization, polarization, &c., to large classes, as easily as to one person. To make this point clear, I will give you a few illustrations. Suppose my subject for consideration today is the circulation of the blood. Here we present to the class a diagramatic representation of the systematic circulation, and point out its course through the vena cava into the right auricle, to the right ventricle, through the aeorta, and through its round of circulation.

Let us now project a microscopic section of the lung; here we see the ultimate capillary net work surrounding the air cells, connecting tissues, etc.; with other specimens we could show the epithelial lining, in fact, follow out all the detail and minutae on the screen as perfectly as though we had our specimens on the stage of the microscope, and we have this advantage: we can describe it to all the class as easily as to one member, and can point out any particular structure, which we can not do when we use the ordinary instrument. We will take another example for illustration: a diagram of a tooth showing the enamel, dentine, etc., and follow with a microscopic specimen. This we can see on the screen almost as plainly as with a diagram. With these illustrations the way is paved for intelligent work with the microscope in the laboratory. When a student mounts a tooth section and places it under the microscope he sees a familiar object. Just what he has seen by the aid of projection and heard described, even to the color and delicate tints of shade. Had the lecturer's illustrations been given on a blackboard instead of by the aid of actual specimens projected on a screen, he would not have recognized them when he came to use actual specimens under the microscope. We will refer to another illustration that can be made very instructive and interesting to a class, namely: the circulation of the capillary net work of a small fish, or foot of a frog. Last winter I projected by the aid of sun light the circulation in the foot of a frog before a class: the capillaries were shown from one-half to two-thirds of an inch in diameter, the red blood corpuscles could be plainly seen coursing through them, with now and then a white one cutting across lots. When the web of the foot was pricked with a needle the vessels congested round the wound, and the wounded end of one capillary projected into the opening (which appeared about three inches in diameter) the blood corpuscles were ejected in the stream with each impulse of the heart; the circulation became slower and slower until the conjestion was complete; the foot was released, rubbed to establish the circulation, and after a short time placed again under the field of the microscope, the circulation was now seen over the entire field except the wounded part. In this short hour we had seen the circulation in the capillaries, the migration of white corpuscles, congestion, a bleeding capillary (showing that the impulse of the heart extends through the small vessels) and the establishing of the circulation by massage after it had ceased. Could any description given have had the vivid impression on those students as the illustration described?

We might continue these illustrations by projecting specimens of every tissue of the body, but we think what has been shown will illustrate our point. As before stated, all branches in science, medicine, and dentistry can be very fully illustrated in this way.

The anatomist can use photographic transparencies of any part of the body and gain much in accuracy and size over the use of charts and drawings, as the object can be greatly magnified and made plain to a large class.

The chemist can use this method of illustration to advantage in the lecture room by projecting reactions, chrystallization, electrolytic action, etc., and thus pave the way for the student to begin his laboratory work with a clear idea of what he is to do.

The physiologist is dependent on illustrations to make his lectures clear, and is obliged to refer to anatomical charts and microscopic specimens.

The teacher of pathology is equally interested in illustrating his work.

We had a fine illustration of this work one year ago at Newport when we listened to Dr. Andrew's lecture and saw his beautiful photo-micrographs of fissures in sections of teeth. All who were present will remember Dr. Sudduth's illustrated lecture "The Products of the Epiblast." I had the pleasure of illustrating this lecture. At its close a prominent physician was asked what he thought of it? He said the illustrations were the most perfect he had ever seen, in following the embryonic tooth from the first illustration to the last. He said "I could almost see that tooth grow."

This method of projection is not confined to transparent objects. Opaque objects as well can be magnified and projected on a screen. The principle is not new, but its application to a projecting microscope we believe is. Some two years ago a scientist desired me to make for him a microscope to project objects by the aid of oblique illuminations. In constructing the instrument I found with a little modification it could be made so as to project opaque objects. Without taking time to describe it, will give a few illustrations of its use.

We will place a tooth on the stage and by the aid of reflection project a clearly defined image on the screen. In this manner any small opaque object can be magnified and plainly shown to a class. Malformations of teeth and roots, pulp nodules, fissures, etc., can be shown by making transverse and longitudinal sections, or any small instrument or mechanical device can also be seen in this way. We believe the teacher of operative dentistry will find in the projection of opaque objects a new field of illustration.

We also believe this method of illustrating by the aid of projection, in its infancy.

There is no want of apparatus, or light for illumination, but there is no complete list of objects, either photographic or microscopic, each teacher is at work in his own way and uses such specimens as are within his reach. We have text books profusely illustrated and systematically arranged. Now what is needed is photo-diagrams, photographic transparencies, and typical microscopic slides, so that the text books and lectures can be fully illustrated on the screen before a class. Drs. Sudduth, Andrews, Hays, and others have done much in this direction, each in his line of work. Others are at work in their specialties. We trust the time is not far distant when typical objects properly arranged in sets, both photographic and microscopic can be as easily obtained as text books on the various scientific subjects. The teacher will then have his notes before him in his illustrations. He can abridge his lectures. This being accomplished the poor student will not feel that he is being talked to death.

